# Takotsubo Cardiomyopathy Following COVID-19 Vaccine Booster Dose: A Case Report

**DOI:** 10.7759/cureus.43295

**Published:** 2023-08-10

**Authors:** Rezaur Rahman Reza, Aalok Parajuli, Tripura Padullaparthi, Swetha Aloori, Anusha Baddam, Aakriti Parajuli, Susannah shiny Karnati, Hira Nasir

**Affiliations:** 1 Internal Medicine, Jalalabad Ragib Rabeya Medical College, Victorville, USA; 2 Medicine and Surgery, Prime Health Care/Desert Valley Hospital, Victorville, USA; 3 Internal Medicine, Malla Reddy Medical College for Women, Hyderabad, IND; 4 Pulmonary and Critical Care Medicine, Johns Hopkins University, Baltimore, USA; 5 Epidemiology and Biostatistics, Benedictine University, Chicago, USA; 6 Internal Medicine, Cebu Doctors University, Mandaue City, PHL; 7 Internal Medicine, Mayo Hospital, Lahore, PAK

**Keywords:** covid-19 vaccine, asthma exacerbation, takotsubo cardiomyopathy, mrna-based vaccination, respiratory failure

## Abstract

Although the efficacy and safety of the coronavirus disease 2019 (COVID-19) vaccine have been established, side effects and adverse events related to the COVID-19 vaccine are still coming out. COVID-19 vaccine also has the potential to cause acute and long-term cardiovascular effects, which include myocarditis, pericarditis, myopericarditis, myocardial infarction, pulmonary embolism, thrombotic thrombocytopenia, and pulmonary hemorrhage. Although uncommon, takotsubo cardiomyopathy (TCM) has also been reported following COVID-19 vaccination. We report a case of TCM following the COVID-19 vaccine in a 59-year-old female who presented with intermittent chest pain and dyspnea following the COVID-19 vaccine booster dose. She had no identifiable triggers for TCM, no risk factors for cardiovascular disease, and normal cardiac enzyme levels, ruling out other causes of cardiac dysfunction. The diagnosis of TCM was supported by imaging findings and the absence of obstructive or thrombotic lesions on angiography.

## Introduction

The coronavirus disease 2019 (COVID-19) pandemic has severely affected the whole globe with dreadful social and economic consequences, eventually controlled with worldwide vaccination. Although the efficacy of the COVID-19 vaccine has been established, side effects and adverse events associated with the COVID-19 vaccine are still coming out [1]. Common side effects of the COVID-19 vaccine include injection site reaction, myalgia, fever, headache, or diarrhea. COVID-19 vaccine also has the potential to cause acute and long-term cardiovascular effects, which include myocarditis, pericarditis, myopericarditis, myocardial infarction, pulmonary embolism, thrombotic thrombocytopenia, and pulmonary hemorrhage [2]. Although uncommon, takotsubo cardiomyopathy (TCM) has also been reported following COVID-19 vaccination [3,4]. The number of TCM cases reported following COVID-19 vaccination is not high [3-5]. Herein, we report a case of TCM induced by the COVID-19 vaccine booster dose.

## Case presentation

A 59-year-old female with a history of hypothyroidism, hyperlipidemia, and celiac disease was brought to the emergency department with sudden onset dyspnea for the last six hours. She also complained of intermittent chest pain for the last two days. The pain was stabbing, progressive with each episode, non-radiating, and worse on exertion with no relieving factors. She received her booster dose of the Moderna COVID-19 vaccine three days ago. She experienced flu-like symptoms after her first dose of the COVID-19 vaccine and was asymptomatic after her subsequent doses. She was compliant with her medications. She had no history of alcohol or substance abuse except for a smoking history for five years, which she quit fifteen years ago.

She was afebrile and oriented to time, place, and person. She had an oxygen saturation of 89% on room air, blood pressure of 150/90 mmHg, and a respiratory rate of 24 breaths/minute. On chest auscultation, she had diffuse lung crepitations with normal heart sounds. The rest of the systemic examination was unremarkable. Arterial blood gas analysis revealed type 1 respiratory failure, and the results of initial investigations are shown in Table 1.

**Table 1:** Initial laboratory evaluations on admission. pH: potential hydrogen, PaCO2: partial pressure of carbon dioxide; PaO2: partial pressure of oxygen.

Parameter	Lab value	Reference range
White cell count	8000 /mm^3^	(4000-11000)
Hemoglobin	11 g/dl	(13-15)
Platelet count	199,000/mcl	(150,000-350,000)
Lactate dehydrogenase	499 IU/l	(1240-222)
Creatine phosphokinase	198 IU/L	(42-152)
Troponin I	21 ng/ml	(0-14)
Serum creatinine	1.1 mg/dl	(0.7-1.3)
Serum calcium	9.9 mg/dl	(9.0-10)
Blood urea nitrogen	19 mg/dl	(07-26)
Alanine aminotransferase	49 IU/L	(8-57)
Alkaline phosphatase	77 mg/dl	(36-95)
C-reactive protein	3 mg/dl	(0.4-1.1)
Erythrocyte sedimentation rate	31/hour	(<21)
Creatine kinase MB	19 ng/ml	(0-05)
Sodium	138 mEq/l	(138-145)
Potassium	3.7 mEq/l	(3.5-4.5)
D-dimer	5.8 ug/ml	(0-1)
pH	7.38	7.35-7.45
PaCO_2_	51 mmHg	35-45
PaO_2_	69 mmHg	75-100

**Figure 1:**
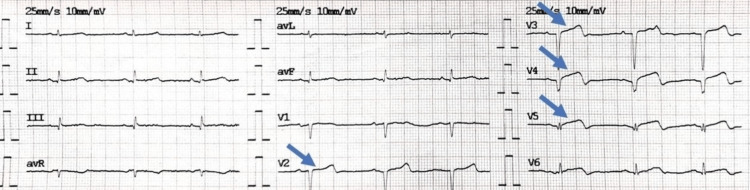
Electrocardiogram revealing ST elevation in leads V2-V5.

**Figure 2:**
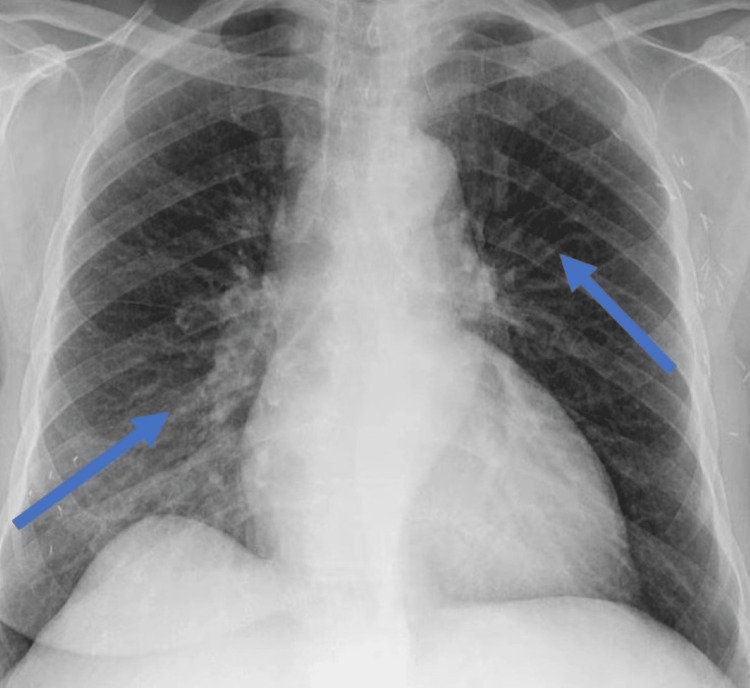
Chest x-ray showing prominent vascular marking suggestive of acute pulmonary edema (blue arrows).

**Figure 3:**
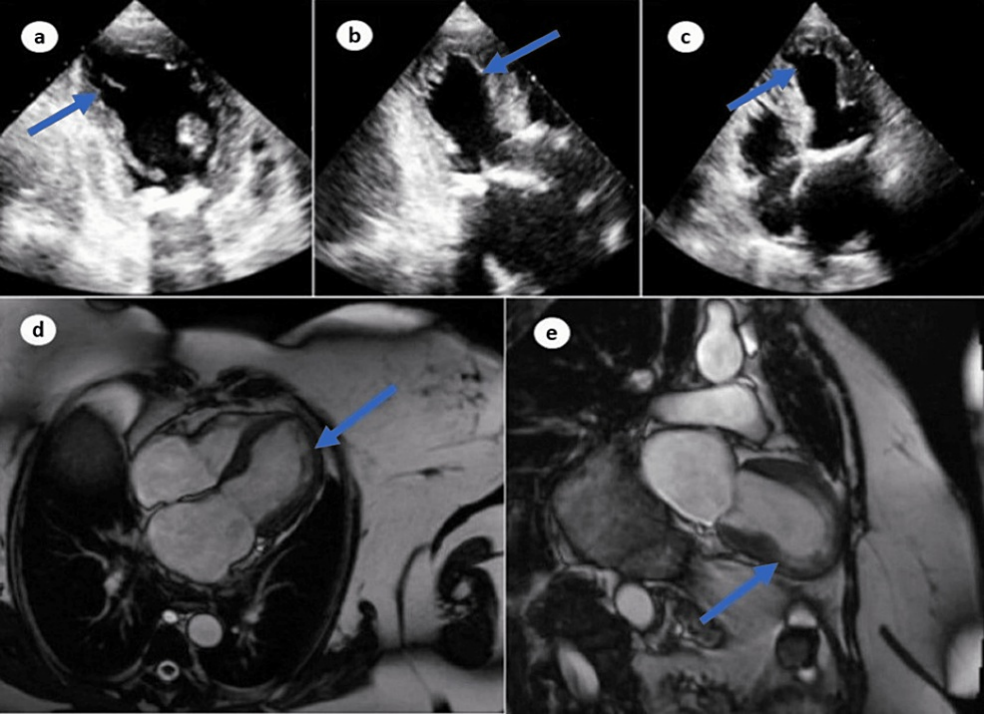
Echocardiogram revealing apical ballooning of heart (a-c) and cardiac ventriculogram revealing apical ballooning ruling out myocarditis (d, e).

**Figure 4:**
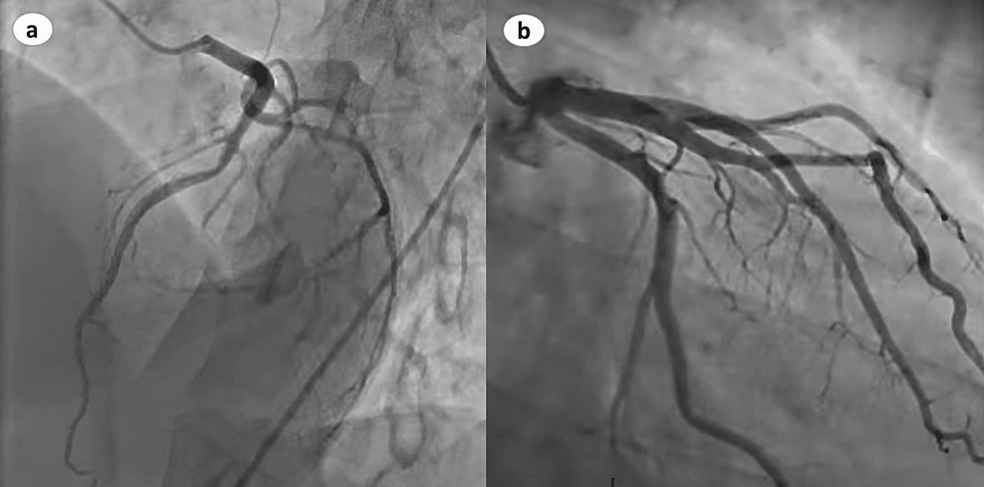
Coronary angiography showing normal right (a) and left (b) coronary arteries.

Her COVID-19 polymerase chain reaction (PCR) was negative. An urgent electrocardiogram (EKG) revealed ST-segment elevation in leads V2-V5 (Figure 1). Chest x-ray was significant for pulmonary edema (Figure 2). Transthoracic echocardiography (TTE) showed diminished left ventricular systolic function with an estimated ejection fraction (EF) of 30% and moderate hypokinesia of the apex and anterolateral wall of the heart (Figure 3). She had persistent tachycardia and fluctuating blood pressure, and due to fluid overload, she became hemodynamically unstable leading to cardiac shock. She was commenced on intravenous norepinephrine and dobutamine due to cardiogenic shock. Chest computed tomography (CT) was performed to rule out pulmonary embolism (PE), which was normal. She was brought to the cath lab urgently and underwent coronary angiography, which was unremarkable, demonstrating no thrombotic or obstructive lesion (Figure 4). Cardiac magnetic resonance imaging revealed apical ballooning of the heart excluding myocarditis (Figure 3). A provisional diagnosis of TCM with a TCM risk score of 61 was made, induced by the COVID-19 vaccine, as no other etiology was identified. Her repeat COVID-19 PCR, including blood culture and viral serology, were normal. Her blood culture, viral serology, and autoimmune screening were unremarkable.

**Figure 5:**
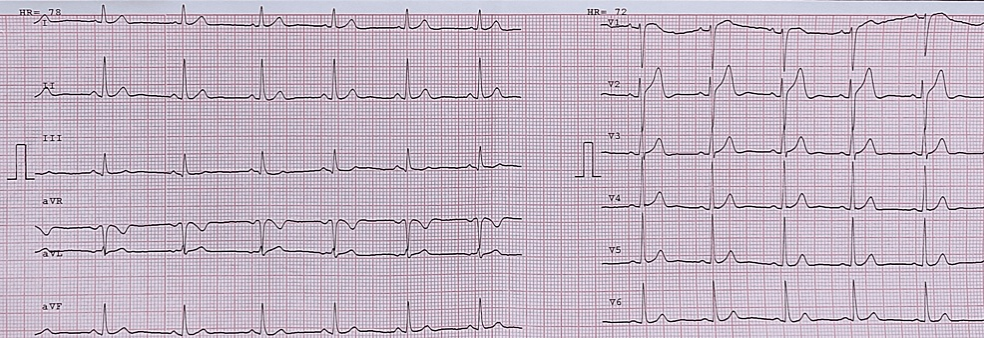
Electrocardiogram revealing unremarkable changes on recent visit.

She was managed with systemic hydrocortisone, systemic furosemide, heparin, and norepinephrine. Her respiratory status improved gradually with the normalization of blood pressure, and she returned to diuresis. An additional dose of furosemide was added, and over the next 36 hours, her norepinephrine dose tapered, and she became hemodynamically stable on day three. Her repeat EKG and TTE were normal on day six, with an improved EF of 55%, and she was discharged on metoprolol due to persistent tachycardia. Her recent follow-up was stable, with a normal EKG (Figure 5).

## Discussion

The symptoms of TCM often mimic those of acute myocardial infarction. Patients may experience chest pain or discomfort, dyspnea, tachycardia or irregular pulse with EKG changes, dizziness, fainting, or low blood pressure, which may indicate coronary artery disease with a raised level of myocardial enzymes in the absence of remarkable coronary artery stenosis on angiography [6]. TCM is relatively uncommon and constitutes around 1% of all patients with suspected acute coronary disease. TCM is primarily seen in females, particularly after menopause [7]. The exact causes of TCM are still not fully understood, but several triggers have been identified. Emotional stressors such as grief, loss, or a sudden emotional shock, such as the death of a loved one or a traumatic event, can initiate the condition. Physical stressors such as surgery, serious illness, asthma attacks, or even extreme physical exertion can also act as triggers [8]. Hormonal imbalances and certain medications have been linked to TCM as well. COVID-19 vaccine, especially the mRNA vaccine, can also potentially cause TCM [9,10]. Although rare, the COVID-19 vaccine is also implicated as an etiology of TCM [11]. A recent analysis highlighted ten cases of vaccine-induced TCM, which include ten female and one male patients. Chest pain was the most presenting symptom, and cases were reported within a week of vaccine administration. All the patients were presented with chest pain or dyspnea and elevated cardiac markers on investigations. EKG changes were dominant in anterior and lateral leads, with an ejection fraction of <50% in all the patients. The length of the hospital stay was not more than two weeks in any patient, and all patients were discharged with no mortality [12].

The pathophysiology of COVID-19 vaccine-induced TCM is unknown; however, several theories have been proposed. COVID-19 vaccines elicit an immune response aimed at producing protective antibodies against the SARS-CoV-2 virus. However, in certain individuals, this immune response may result in an exaggerated inflammatory cascade, leading to endothelial dysfunction, microvascular dysfunction, and myocardial injury. The release of pro-inflammatory cytokines, such as interleukin-6 (IL-6), may contribute to the pathophysiology of TCM [13]. The autonomic nervous system plays a crucial role in regulating cardiac function. Excessive sympathetic nervous system activation and reduced parasympathetic tone have been implicated in the pathogenesis of TCM due to the excessive release of catecholamines. The stress associated with COVID-19 vaccination could potentially dysregulate the autonomic nervous system, contributing to the development of cardiac dysfunction [14]. Endothelial dysfunction and impaired coronary microvascular function have been observed in TCM. COVID-19 vaccines may induce transient endothelial dysfunction and microvascular abnormalities, impairing coronary blood flow and subsequent myocardial dysfunction [15].

Our patient presented with dyspnea and chest pain after COVID-19 vaccination and was diagnosed with TCM. She had no identifiable trigger for TCM, including stress, emotional and physical trauma. Her COVID-19 PCR was negative. Moreover, she had no identifiable risk factor for cardiovascular disease, normal creatine kinase level, and absence of obstructive or thrombotic lesion or coronary angiography in the presence of EKG, and echocardiography findings justify that ischemic heart disease was unlikely. Massive immune response after the COVID-19 vaccine may have contributed to the development of TCM.

## Conclusions

It is important to note that while there have been rare cases of TCM following COVID-19 vaccination, the overall incidence remains extremely low. The benefits of COVID-19 vaccination in preventing severe illness and complications far outweigh the potential risks. It is essential for physicians to be aware of the potential cardiovascular effects of COVID-19 vaccines, including the rare occurrence of TCM. Prompt recognition and appropriate management of TCM are crucial to ensure favorable outcomes for patients. Further research is warranted to understand the mechanisms underlying vaccine-induced TCM better and identify individuals at higher risk.
